# Preclinical evaluation of a nanoformulated antihelminthic, niclosamide, in ovarian cancer

**DOI:** 10.18632/oncotarget.7113

**Published:** 2016-02-01

**Authors:** Chi-Kang Lin, Meng-Yi Bai, Teh-Min Hu, Yu-Chi Wang, Tai-Kuang Chao, Shao-Ju Weng, Rui-Lan Huang, Po-Hsuan Su, Hung-Cheng Lai

**Affiliations:** ^1^ Graduate Institute of Medical Sciences, National Defense Medical Center, Taipei, Taiwan; ^2^ Department of Obstetrics and Gynecology, Tri-Service General Hospital, Taipei, Taiwan; ^3^ Graduate Institute of Biomedical Engineering, National Taiwan University of Science and Technology, Taipei, Taiwan; ^4^ School of Pharmacy, National Defense Medical Center, Taipei, Taiwan; ^5^ Department of Pathology, Tri-Service General Hospital, National Defense Medical Center, Taipei, Taiwan; ^6^ Department of Biology and Anatomy, National Defense Medical Center, Taipei, Taiwan; ^7^ Department of Obstetrics and Gynecology, Shuang-Ho Hospital, Taipei Medical University, New Taipei City, Taiwan; ^8^ Department of Obstetrics and Gynecology, School of Medicine, College of Medicine, Taipei Medical University, Taipei, Taiwan

**Keywords:** nanoformulation, nanomedicine, niclosamide, ovarian cancer, cancer metabolism

## Abstract

Ovarian cancer treatment remains a challenge and targeting cancer stem cells presents a promising strategy. Niclosamide is an “old” antihelminthic drug that uncouples mitochondria of intestinal parasites. Although recent studies demonstrated that niclosamide could be a potential anticancer agent, its poor water solubility needs to be overcome before further preclinical and clinical investigations can be conducted. Therefore, we evaluated a novel nanosuspension of niclosamide (nano-NI) for its effect against ovarian cancer. Nano-NI effectively inhibited the growth of ovarian cancer cells in which it induced a metabolic shift to glycolysis at a concentration of less than 3 μM *in vitro* and suppressed tumor growth without obvious toxicity at an oral dose of 100 mg/kg *in vivo*. In a pharmacokinetic study after oral administration, nano-NI showed rapid absorption (reaching the maximum plasma concentration within 5 min) and improved the bioavailability (the estimated bioavailability for oral nano-NI was 25%). In conclusion, nano-NI has the potential to be a new treatment modality for ovarian cancer and, therefore, further clinical trials are warranted.

## INTRODUCTION

Ovarian cancer is the most lethal gynecologic malignancy in the world [1]. Most patients undergo cytoreductive surgery and receive a combination of paclitaxel and platinum-based chemotherapy. However, numerous patients are diagnosed at an advanced stage and, therefore, have a poor prognosis due to distant metastases and chemoresistance [2]. Although paclitaxel represents a breakthrough in the treatment of ovarian cancer, the overall 5-year survival rate of patients with stage III disease is still approximately 40% [2–4]. Furthermore, the inclusion of targeted therapy for ovarian cancer offers limited therapeutic advantages [5, 6]. The recently approved antiangiogenesis agent, bevacizumab, also showed a limited improvement in progression-free survival (PFS) [7–9]. Therefore, there is an urgent need to develop novel, effective, and safe agents for ovarian cancer treatment.

Targeting cancer stem cells is an emerging concept in cancer therapy [10]. Ovarian cancer stem cells play an important role in chemoresistance and cancer recurrence [11]. In particular, several “old drugs” have been used in drug repurposing studies to inhibit cancer cell growth by altering the metabolic pathways of cancer cells [12, 13]. We previously used a cancer stem cell-based drug screening platform to demonstrate that niclosamide, an old and safe antihelminthic drug, inhibits the growth of tumor-initiating cells by modulating their metabolic signaling pathways [14, 15]. Furthermore, recent studies indicate that niclosamide exhibits anticancer effects against various human cancer cells by acting on multiple cell signaling pathways and inducing mitochondrial uncoupling [16–21]. Therefore, niclosamide is considered an “old drug” with potential for inclusion in new multitargeted therapy for cancer [17].

Niclosamide has a poor water solubility of 0.23 μg/mL [22] and, therefore, has low systemic bioavailability (∼10%) when administered orally, which is beneficial for treating local parasitic infections of the intestines while minimizing systemic exposure [22]. However, poor solubility is an obstacle to the further clinical development of niclosamide as a novel anticancer drug. One approach to improving drug solubility is a chemical modification, and several water-soluble niclosamide derivatives have been synthesized [23]. However, these derivatives are new chemical entities that require comprehensive preclinical and clinical studies to confirm their efficacy and safety. Another approach is to reduce the particle size of an approved drug by using emerging nanotechnology [24–27]. This formulation-based approach is believed to streamline the drug development process because the pharmacological and toxicological profiles are already known. We have successfully fabricated a nanosuspension of niclosamide (nano-NI) with a greatly enhanced aqueous dissolution rate, owing to its significantly reduced particle size [28]. The novel nano-NI could have clinical applications in the treatment of ovarian cancer; however, before clinical studies, preclinical efficacy, toxicity, and pharmacokinetic as well as pharmacodynamic studies are required.

In this study, we evaluated the *in vitro* and *in vivo* activity and toxicity of nano-NI in cell and animal models as well as the effects of the novel formulation on water solubility and ovarian cell metabolism. Then, we determined the oral bioavailability and pharmacological effects of the new formulation. The information obtained from the present study should provide a foundation for future clinical investigations.

## RESULTS

### Nano-NI suppresses ovarian cancer cell proliferation

**Figure 1:**
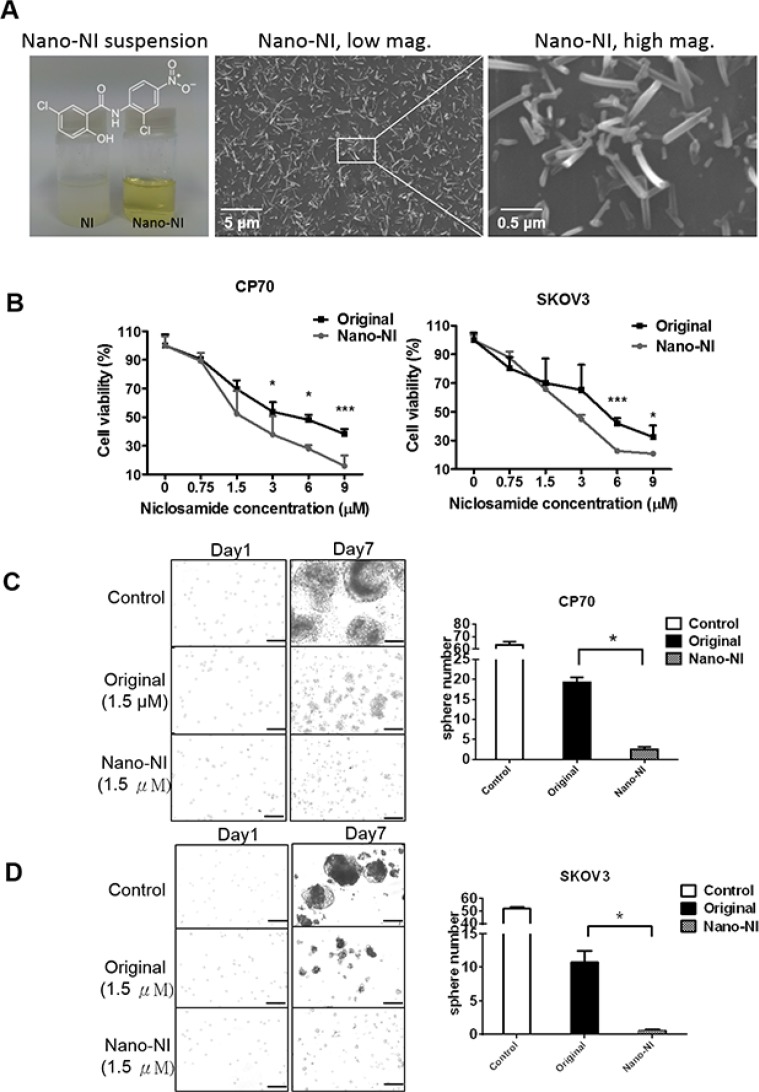
Nano-NI suppresses ovarian cancer cell proliferation more efficiently than the original form of niclosamide *in vitro* (**A**) Nano-NI was more soluble than its original form. The nano form and original form of niclosamide dissolved in PBS at the same concentration (455.8 μg/L) with equal volumes. The chemical structure of niclosamide is shown (left). Nano-NI particles are shown in the scanning electron microscopy image at low magnification (20 kV, × 5000; scale bar *=* 5 μM; middle) and at high magnification (20 kV, × 30,000; scale bar *=* 0.5 μM; right). (**B**) Nano-NI inhibits cell proliferation more efficiently than the original form of niclosamide in ovarian cancer cell lines CP70 and SKOV3; cells were treated with different forms of niclosamide at various concentrations (0, 0.75, 1.5, 3, 6, and 9 μM) for 72 h, and cell viability was measured by using the MTS assay. Values are expressed as the percentage of viable cells, with the control cells set at 100%. The results represent the mean ± standard deviation of triplicate samples. (**C, D**) The comparison of sphere formation inhibition between nano-NI and original niclosamide treatment using CP70 and SKOV3 ovarian cancer cells (control, original niclosamide and nano-NI). Bar,100 μm. Statistical significance (*p*) was determined by using the Student *t* test (*p < 0.05, **p < 0.01, ***p < 0.001).

In the present study, a colloid dispersion of nanosized NI in PBS stabilized with 1% polyvinyl alcohol (PVA), was prepared using an electrospray procedure [28]. While the coarse suspension of the original niclosamide was turbid, the nano-NI colloidal dispersion was almost transparent, with a yellowish color (Figure 1A). The scanning electron microscope images revealed the nanosuspension morphology, with an average particle diameter and length of 105 ± 21 and 493 ± 151 nm (Figure 1A). To investigate the inhibitory effect of the nano-NI on the growth of ovarian cancer cells, CP70 and SKOV3 cells were treated with the nano-NI and original niclosamide formulations for 72 h. Nano-NI was found to be significantly more cytotoxic to ovarian cancer cells than the original form was, with IC_50_ values of 3.59 and 3.38 μM for CP70 and SKOV3 cells, respectively (Figure 1B). The nano-NI demonstrated significantly higher inhibitory effects on sphere formation than the original niclosamide did (Figure 1C and 1D).

### Nano-NI disrupts ovarian cancer cell metabolism

**Figure 2:**
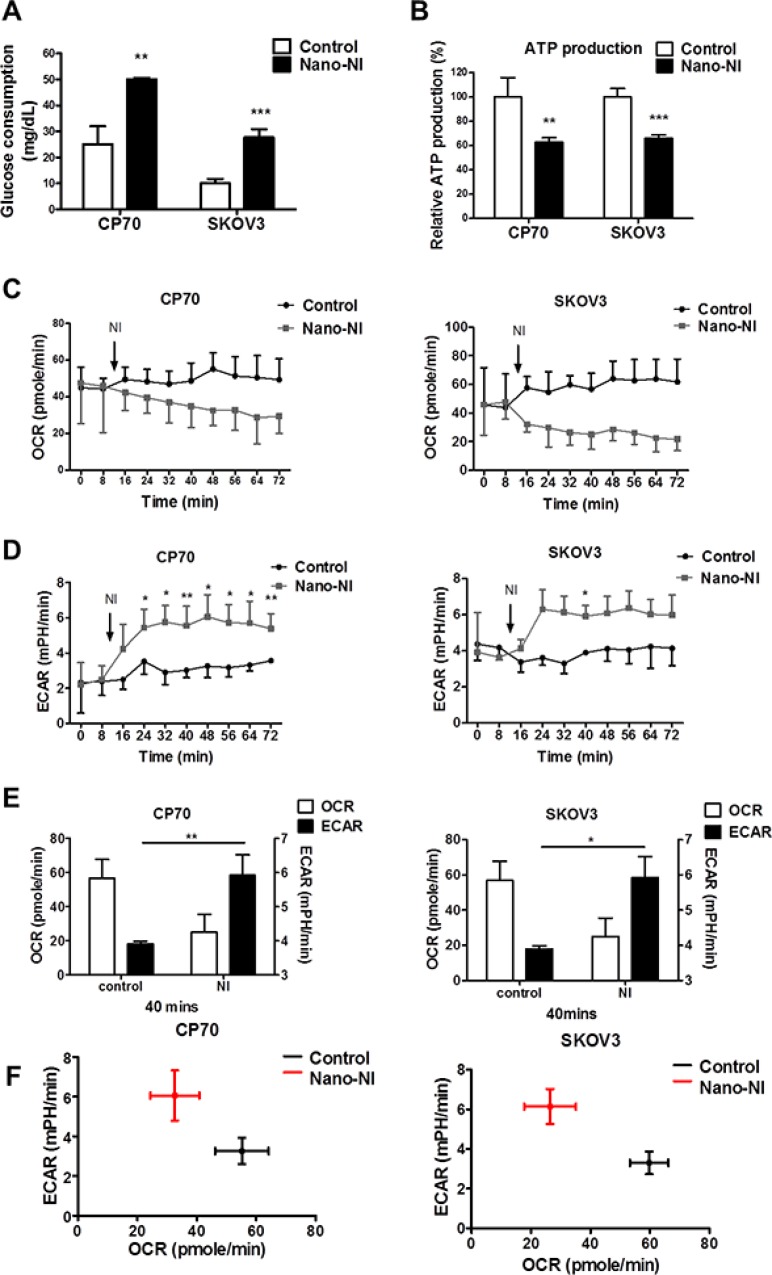
Nano-NI changes the ovarian cancer metabolic phenotype and prompts a metabolic shift (**A**) Glucose consumption increased in both CP70 and SKOV3 ovarian cancer cell lines (−/*+* nano-NI) after 4 h of treatment, (*n =* 3; 2 × 10^5^ cells/well, *p =* 0.01 in the CP70 group, *p =* 0.002 in SKOV3). (**B**) In the ATP production assay of CP70 and SKOV3 cells (−/*+* nano-NI), cellular ATP levels were measured using a luciferase-based assay kit and normalized to controls, revealing that nano-NI inhibited ATP production in both cell lines (*p =* 0.008 in CP70 group, *p =* 0.0008 in SKOV3 group). (**C**) Nano-NI induces a decreasing trend in the cell respiratory rate (OCR) in both CP70 and SKOV3 cells. (**D**) Nano-NI induces a progressive increasing trend in the cell glycolysis rate (ECAR) in both CP70 and SKOV3 cells. (**E**) OCR decrease and ECAR increase after nano-NI treatment in both CP70 and SKOV3 cells after 40 min of treatment. (**F**) Nano-NI induced a shift of OCR/ECAR in CP70 and SKOV3 cells (from the black cross to the red cross). (*p < 0.05, **p < 0.01, ***p < 0.001 versus the corresponding control value).

To explore the mechanism underlying the nano-NI-induced alteration of the ovarian cancer cell metabolic phenotype, we measured glucose consumption in ovarian cancer cell lines. The glucose consumption by the control and nano-NI groups was 25 ± 7 and 50 ± 0.57 mg/dL, respectively in CP70 cells and 10 ± 1.7 and 27.7 ± 3.2 mg/dL, respectively, in SKOV3 cells (Figure 2A). The nano-NI-treated group cells took up more glucose than control cells did. To determine whether nano-NI affected ovarian cancer cell energy production, we performed an ATP production assay with or without nano-NI treatment. The nano-NI decreased ATP production by 37 and 34% in CP70 and SKOV3 cells, respectively (Figure 2B). The increased uptake of glucose with decreased ATP production after nano-NI treatment suggests that the cancer cells shifted to an inefficient energy production mode. To assess the effect of nano-NI on the ovarian cancer cell metabolic shift, we used a flux analyzer (XF24 Extracellular Flux Analyzer, Seahorse Bioscience, North Billerica, MA, USA) to measure the oxygen consumption rate (OCR) and the extracellular acidification rate (ECAR) of the control and nano-NI-treated groups. The ovarian cancer cells treated with nano-NI showed a decrease in the OCR and a compensatory increase in the ECAR in both CP70 and SKOV3 cells (Figure 2C–2E). The shift from oxidative phosphorylation to aerobic glycolysis was observed in both ovarian CP70 and SKOV3 cancer cell lines (Figure 2F). In summary, the nano-NI formulation decreased the metabolic activity of ovarian cancer cells and caused a metabolic shift from oxidative phosphorylation to glycolysis.

### Oral nano-NI inhibits ovarian cancer growth *in vivo*

**Figure 3:**
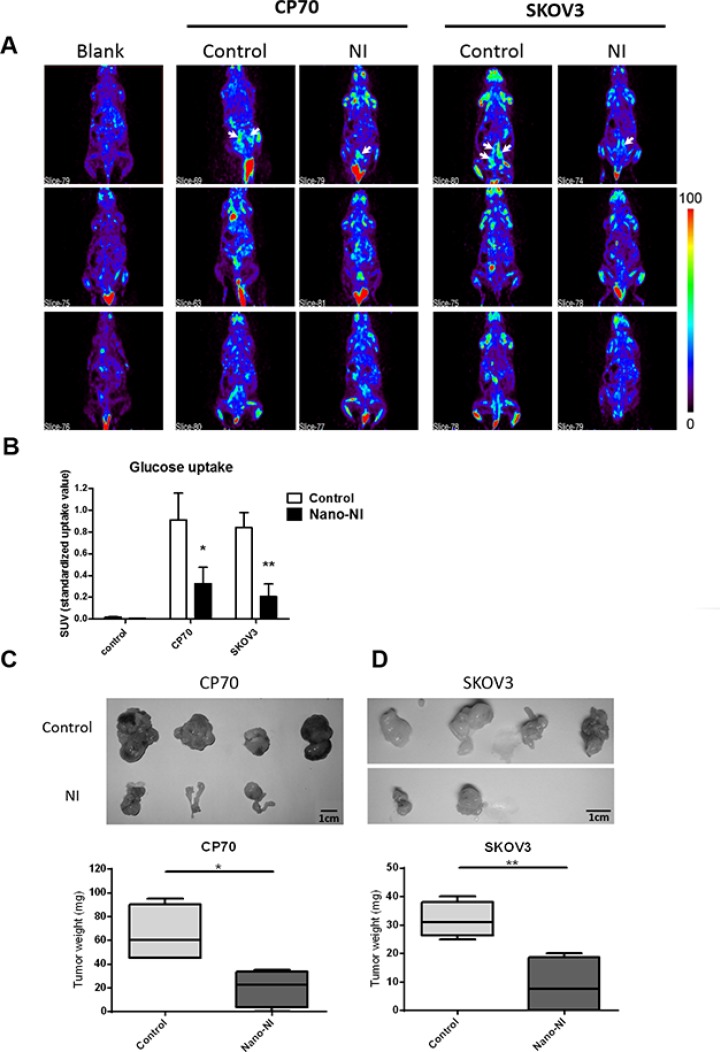
Nano-NI suppressed ovarian cancer tumor growth in NOD/SCID mice (**A**) Animal-PET images were acquired 30–60 min after tail vein injection of 0.2–0.25 mCi [^18^F]-FDG in the blank imaging group (negative control), control group (positive control, oral PBS), and niclosamide group (oral nano-NI); the tumor sites are indicated by the white arrows. (**B**) SUVs of the blank, control, and nano-NI treated groups of CP70 and SKOV3 cells (values shown as mean ± standard deviation). (**C, D**) The growth of tumors from ovarian cancer cell lines (CP70 and SKOV3) treated with nano-NI compared with control groups reveal the inhibitory effect of nano-NI on tumorigenicity (*p < 0.05, **p < 0.01, versus corresponding control values).

To further assess the efficiency of nano-NI in targeting ovarian cancer *in vivo*, we used non-obese diabetic (NOD)/severe combined immunodeficient (SCID) mice in a xenograft experiment in which each group of mice was injected with 10^6^ tumor cells intraperitoneally. Nano-NI (100 mg/kg) was administered via oral gavage five times a week. Positron emission tomography (PET) scan analysis of the tumor growth after 2 weeks of treatment showed significant differences between the control and drug-treated groups (Figure 3A). The mean standardized uptake value (SUV) of the nano-NI-treated group was significantly lower than that of the CP70 cell control group was (0.32 ± 0.14 mg/dL and 0.91 ± 0.24 mg/dL, respectively, Figure 3B). The values were consistent with those obtained in SKOV3 cells, which were 0.20 ± 0.11 and 0.83 ± 0.13 in the oral nano-NI and PBS-treated control groups, respectively. The mice were euthanized 3–4 weeks after the experiment commenced. The tumor sizes are shown in Figure 3C while the tumor weights of the treatment group were significantly reduced (Figure 3D). Therefore, we demonstrated the efficacy of a new oral nanomedicine in an ovarian cancer model.

### Toxicity evaluation of nano-NI *in vivo*

**Figure 4:**
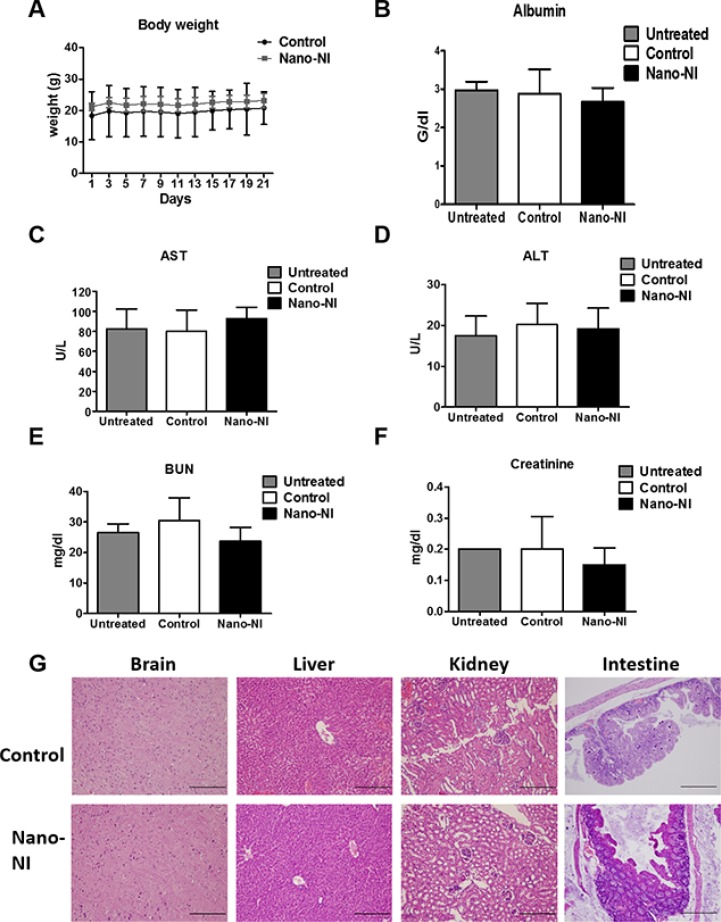
Toxicity analysis in mice treated with nano-NI (**A**) Weight records of NOD/SCID mice in the PBS control and nano-NI-treated groups, from day 1 to 23. (**B**) Albumin level of normal control, PBS control, and nano-NI groups of NOD/SCID mice after the experiment. (**C, D**) Hepatic function of the normal control, PBS control, and nano-NI groups of NOD/SCID mice after 3–4 weeks of the experiment. (**E, F**) Renal function analyses of the normal control, PBS control, and nano-NI groups of the NOD/SCID mice. (**G**) H & E staining of the vital organs of both PBS control and nano-NI groups of the NOD/SCID mice; there were no pathological changes in tissue appearance in either group (scale bar *=* 100 μm).

To explore the toxicity of nano-NI, we monitored the general condition of the mice. The body weights were comparable at 26.1 ± 1.6 and 25.6 ± 1.0 g in the PBS control and nano-NI (100 mg/kg) groups, respectively (Figure 4A). After 4 weeks of the experiment, the mice were euthanized for tumor collection and blood sampling for drug toxicity analyses. The control and orally treated nano-IN groups did not show significant differences, respectively, in nutrition levels (albumin, 2.9 ± 0.6 vs. 2.7 ± 0.4, Figure 4B), renal function (blood urea nitrogen [BUN], 30 ± 7.4 vs. 23 ± 4.0 and creatinine, 0.2 ± 0.1 vs. 0.15 ± 0.05, Figure 4C and 4D), and hepatic function (aspartate aminotransferase [AST], 80 ± 21 vs. 92 ± 10 and alanine aminotransferase [ALT], 2 0 ± 5.2 vs. 19.2 ± 5, Figure 4E and 4F). Histopathological analysis of tissues from the vital organs (the brain, kidney, intestine, and liver) revealed no damage in either group (Figure 4G). In addition to these tests, there were no significant differences in blood cell counts, hemoglobin, and platelet counts between the control and nano-NI groups (Supplementary Figure S1). This toxicity evaluation showed that oral nano-NI had no toxic effect on either group of mice in terms of weight, plasma albumin levels, and blood cell counts, and revealed no adverse effects on vital organ function in the rodents, which suggests that nano-NI is safe for animals.

### Bioavailability of nano-NI after oral administration to sprague-dawley rats

**Figure 5:**
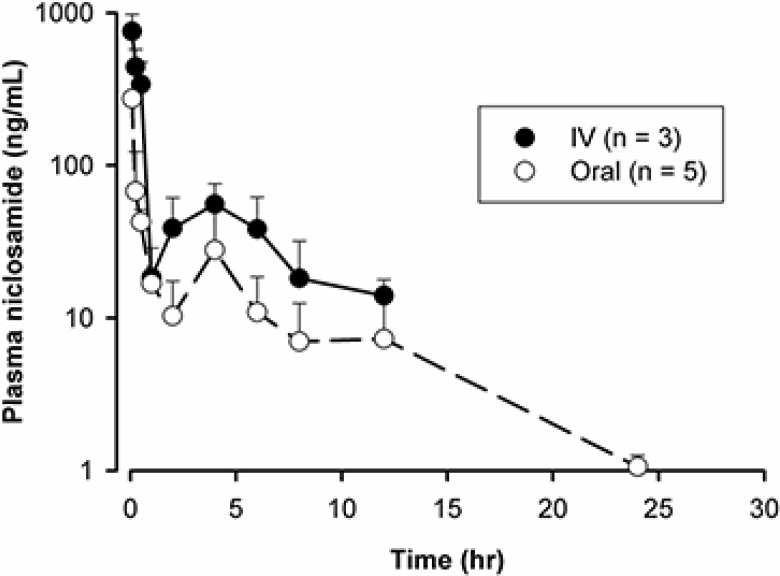
Pharmacokinetic analysis of nano-NI after IV and oral administration (2 mg/kg IV, n = 3; 5 mg/kg orally, n = 5) in rats Niclosamide concentrations were quantified by LC/MS-MS and data are shown as ng/mL.

To evaluate the bioavailability of nano-NI after oral administration, we performed a pharmacokinetic study using high-performance liquid chromatography (HPLC) coupled with liquid chromatography/tandem mass spectrometry (LC-MS/MS). The dosing solution, prepared by dissolving nano-NI in PBS, was administered to female Sprague-Dawley rats by oral gavage or intravenous (IV) injection (5 and 2 mg/kg, respectively). Figure 5 shows the plasma concentration-time profiles of niclosamide. After oral administration, the peak concentration of niclosamide was observed at the first sampling time (5 min), followed by a fast distribution phase. The data indicate that the absorption of niclosamide was rapid such that the absorption phase, typically observed in the pharmacokinetic profile following the oral administration of drugs, was not observed. To verify the peak absorption time, an additional pharmacokinetic study was conducted with more intensive sampling at early time points. The peak appeared again at the first sampling time at 1 min (for both oral and IV routes, Supplementary Figure S2), suggesting that the drug reached the systemic circulation almost instantaneously following oral administration of the solution. It is interesting to note that the plasma concentration peaked right after the distribution phase at 4 h for both the oral and IV routes (Figure 5). This finding was consistent with that of a previous study in mice [16]. Considering that a second peak was also observed with the IV route, this phenomenon may be attributable to an enterohepatic recycling mechanism [29]. As shown in Figure 5, the mean area under the concentration-time curve (AUC) was calculated to be 669.5 and 1058 h/(ng·mL) for the oral and IV routes, respectively. Therefore, the estimated bioavailability for oral niclosamide was 25%.

## DISCUSSION

Considering the widespread incidences of drug resistance and toxicity, as well as selectivity for anticancer therapy, old drugs remain a strong potential source of alternative agents for cancer therapy. Recently, drug-repurposing studies have presented a promising strategy for discovering new therapeutic agents for cancer treatment. The concept of repurposing existing drugs for use in cancer treatment was described previously, and successful examples include sodium stibogluconate. This drug has been used for nearly 60 years to treat visceral leishmaniasis in humans and was considered druggable for combined use with interferon in cancer therapy in a phase I study [30]. Other successful examples are the antimalarial drug artesunate, which has proven effective in treating human renal cell carcinoma alone or in combination with other therapeutic agents [31]. Successful translation of repurposed drugs for clinical use requires thorough *in vitro* and *in vivo* preclinical investigations. Moreover, during the drug development process, it is essential to understand the physicochemical properties of a new molecular entity and to develop a suitable formulation for drug administration and delivery. Similar to drugs discovered through traditional pathways, a repurposed “old” drug may face some formulation problems that need to be addressed.

The drug niclosamide was approved for intestinal parasite infection and has low water solubility and oral bioavailability. The drug has been found to exert growth inhibitory effects against many cancer cells by acting on multiple pathways of cancer cell metabolism. Previously, we showed the potential anti-ovarian cancer activity of niclosamide. In the present study, for the first time we report the preclinical efficacy, toxicity, and pharmacokinetic properties of an orally administered niclosamide nanosuspension (nano-NI) for ovarian cancer treatment. Our findings indicate that nano-niclosamide suppressed ovarian cancer cell growth more efficiently than the original form did *in vitro* and inhibited energy production in ovarian cancer cells, leading to a functionally altered metabolic phenotype. Furthermore, our data also show that oral administration of nano-NI inhibits ovarian tumor growth with minor hepatic and renal toxicity in mice. In addition, nano-NI exhibits improved oral bioavailability of niclosamide.

Nanotechnology is an area of science devoted to manipulating particle size to obtain particles in the nanometer scale range of 1–100 nm (or sometimes up to 1000 nm). Nanosized materials have unique physicochemical properties such as a large surface area to mass ratio as well as high permeability and solubility, which can be used to overcome the limitations of traditional therapeutic and diagnostic agents [32]. Recently, nanodrug delivery systems (nano-DDS) have been extensively studied to improve cancer treatment [33]. Nano-DDS currently in clinical use include liposomes, nanoemulsions, lipid nanocarriers, and micelles [34]. Liposomal doxorubicin is the best-known nanoparticle formulation for metastatic ovarian cancer therapy. For solid tumor treatment, higher tumor accumulation is anticipated with nanoparticles because of the enhanced permeation and retention effect caused by the leaky nature of the tumor vasculature [35]. Although liposomal doxorubicin offers an advantage of reduced cardiac toxicity, its use is limited by neurotoxicity and hand-foot syndrome [36, 37]. In addition, liposomal doxorubicin has technological and manufacturing issues that limit its large-scale production and supply [38]. The use of the liposomal formulations is still limited owing to the possible promotion of tumor angiogenesis and inhibition of tumor immunologic response [39]. In this study, a directly fabricated nano-NI in PBS was investigated without an adjuvant, which may be safer for clinical application.

Several cosolvents have been used to formulate niclosamide solutions including polyethylene glycol (PEG), cremophor, and dimethyl sulfoxide (DMSO) [16–18, 40]. All of these cosolvents show dose-dependent toxicities. For example, a recent report revealed that the long-term uptake of DMSO results in brain degeneration and widespread apoptosis in the developing central nervous system [41]. Previous studies on the oral administration of niclosamide successfully proved that it has antitumor activity against colon cancer *in vivo*, but the studies were not without limitations. In the present study, we found that nano-NI could be dispersed in the PBS-based dosing solution without the addition of organic solvents. The nano-NI overcame the poor water-solubility of niclosamide by minimizing its particle size, thereby increasing the surface area of the original powder without altering its chemical structure [25]. The reduced use of a nonaqueous solvent is advantageous for further developing clinically acceptable formulations. Our nano-NI with a nanosized suspension in PBS is a pure agent without cosolvent toxicity.

To improve the treatment of ovarian cancer, it is important to include new anticancer drugs that show less toxicity, increased ease of production, and can be administered orally to the currently available treatment options. Niclosamide may be a next-generation anti-ovarian cancer drug because it targets cancer cell metabolism and has a good safety profile. The drug is used for treating parasitic intestinal infections; therefore, its site of action after oral administration is in the intestine. Its low aqueous solubility and poor oral bioavailability are advantageous for its antiparasitic effect. Moreover, the nanosized drug may exhibit better oral bioavailability. In the present study, the oral bioavailability of our nano-NI solution was 25%, which is a significant improvement over the reported value of 10% [23]. However, as a potential drug for ovarian cancer treatment, the bioavailability of niclosamide needs to be improved, and its pharmacokinetic properties, as well as efficacy and toxicity in the context of improved absorption, should be characterized.

Considering the unique biology of cancer cells, targeting their metabolism may be a possible therapeutic strategy [41, 42]. Metabolism comprises the various cellular chemical reactions that sustain life and is different in cancer cells from that in normal cells. Therefore, metabolism and could be a useful therapeutic target [43]. For example, metformin and statins have been associated with improved survival in cancer, which may be attributed to their effects on glycolytic pathways and lipid metabolism of cancer cells, respectively [12, 44–46]. In this study, the metabolic interruption induced by nano-NI may be one of the mechanisms underlying its effects against ovarian cancer.

Niclosamide is approved by the US Food and Drug Administration (FDA) as an antihelminthic drug and has been recently investigated for use in cancer therapy. Fundamentally, the mechanism of action of niclosamide involves uncoupling of the mitochondria of target cells. The mitochondrion is vital for cellular bioenergetics and plays a central role in the apoptotic process. Several lines of evidence indicate that niclosamide inhibits tumor cell growth by interrupting multiple pathways (Wnt, Notch, STAT3, NF-κB, and mTORc1) and the generation of reactive oxygen species in several cancer cells [16–18, 42–44]. Therefore, metabolic intervention by old drugs could be a new approach to cancer therapy [45–47]. From the clinical point of view, cancer metabolism has been proposed as a therapeutic target in ovarian cancer [48, 49]. In a previous study, we showed that niclosamide downregulates several essential metabolism-associated genes, and we proposed that it could inhibit ovarian cancer cells by interfering with metabolic pathways [14]. In the present study, we show that nano-NI changed the phosphorylation-to-glycolysis ratio of cancer cells, and suppressed tumor growth, thereby providing evidence supporting interference with metabolism as a potential approach to therapy. Cancer cells possess a unique metabolic status known as the Warburg effect, which depends on aerobic glycolysis and diverges significantly from that of normal cells [50]. The metabolic alteration in tumor cells may confer sensitivity to some targeted therapies and chemotherapeutic agents [50, 51]. Therefore, our results support the testing of a combination of nano-NI with these agents to improve cancer therapy.

The incorporation of this nanosuspension of an old drug into current chemotherapy guidelines is a challenge. The current standard therapy for ovarian cancer includes taxanes and platinum-based chemotherapy after cytoreductive surgery. Among treated patients, nearly 70 to 80% will experience disease recurrence [2, 52]. For patients with platinum-sensitive recurrent ovarian cancer, chemotherapy with combined paclitaxel-carboplatin has been shown to lead to a longer overall survival (OS) than with platinum alone, but with more side effects [52]. Combination therapy in patients with platinum-sensitive recurrent ovarian cancer using carboplatin and gemcitabine, with or without bevacizumab, may prolong progression-free survival (PFO), but not OS [53, 54]. For patients with platinum-resistant ovarian cancer, the use of single agents such as the taxanes has only a 22% response rate while pegylated liposomal doxorubicin and gemcitabine may have a response rate of 33% [55, 56]. Combined chemotherapy may not prolong OS due to incidences of more side effects than occurs with single-agent chemotherapy in patients with platinum-resistance. Owing to its safety following oral use, the future application of nano-NI combined with other chemotherapeutic agents may warrant further investigation. In the future, designs of clinical trials using nano-NI alone or in combination with other chemotherapeutic agents will lead to the clinical application of this newly formulated old drug.

In conclusion, the newly developed nanosuspension of niclosamide without adjuvants was water soluble, effective, and safe in this preclinical study. Therefore, future clinical evaluation is warranted.

## MATERIALS AND METHODS

### Cell lines

Two human ovarian cancer cell lines were used in this study. They were the SKOV3, which was purchased from the American Type Culture Collection (ATCC, Manassas, VA, USA) and CP70, from Dr. Tim Huang's Laboratory (University of Texas Health Science Center, San Antonio TX, USA). CP70 and SKOV-3 cells were maintained as monolayer cultures in RPMI-1640 medium (Biological Industries Inc.) supplemented with 10% fetal bovine serum (Gibco), 0.1% NaHCO_3_ (Sigma-Aldrich), 0.1% *N*-2-hydroxyethylpiperazine-*N*-2′-ethanesulfonic acid (Sigma-Aldrich), 1% non-essential amino acids (NEAA), 1% sodium pyruvate, and 0.1% penicillin-streptomycin (all from Biological Industries Inc.) at 37°C in a 5% CO_2_ atmosphere. SKOV3 and CP70 cells were passaged every 3 days for a maximum of 20 passages.

### Preparation of nano-NI using single-capillary electrospray

The setup of the single-capillary electrospray system has been described previously [28]. For the electrospray processing, a stock solution of niclosamide was prepared by dissolving 5 mg in 2.5 mL of a cosolvent (acetonitrile/DMSO, 1/4, *v*/*v*). The collection substrate was a petri dish filled with PBS solution containing 1% (*w*/*v*) polyvinyl alcohol (PVA) as a stabilizer (i.e., 1:1 mixture of a 2% [*w*/*v*] PVA solution and 2 × PBS solution). The capillary tube and collection substrate were approximately 3 cm apart, and the applied voltage ranged from 5 to 7 kV. The collection time was approximately 13.5 h, during which a nanosuspension containing a maximum niclosamide concentration of 455.8 μg/mL could be obtained. The electrospray modes of the system were monitored by viewing the liquid meniscus at the exit of the capillary tube. The meniscus was illuminated with a diffusing light, and its shape was observed using a microscope system that consisted of a microscope lens (Retro Zoom 65, Model: 30–43–10), a digital camera (STC-620PWT, Sentech), and a high-resolution monitor. The spray jet was recorded using a computer. The solvents were evaporated during the electrospray process. The prepared nano-NI formulations were further diluted to the desired concentrations for use in subsequent cell and animal studies. The UV-visible spectrum indicated that the previously described organic solvents used for producing the nano-NI were almost completely evaporated during electrospray processing (Supplementary Figure S3).

### Cell proliferation and chemosensitivity assay

CP70 and SKOV3 cell viabilities were detected using the MTS cell proliferation assay (Promega). Briefly, CP70 and SKOV3 (1000 cells each) were seeded in 96-well plates containing 100 μL media/well overnight. Then, they were exposed to nano-NI or niclosamide for 72 h. The MTS solution was prepared from the CellTiter 96R aqueous MTS reagent powder and kept at −20°C for long-term storage, and at 4°C away from light before use. Next, 20 μL of the MTS solution was added to each well, where the viable cells generated soluble formazan. After an incubation period of 45 to 60 min, the fluorescence absorbance at 490 nm was measured using a 96-well microplate reader (BioTek). Each reaction was assayed at least thrice. The results are expressed as the ratio of the absorbance of each sample to that of the untreated samples with media alone. The proliferation assays were performed in triplicate. All *in vitro* studies were conducted in triplicate in two independent experiments in the different cell lines.

### Sphere formation assay

CP70 and SKOV3 ovarian cells were cultured in ultra-low attachment plates (Corning). The culture medium used was high glucose Dulbecco's modified Eagle's medium (DMEM) plus GlutaMAX^™^-I containing 5% FBS suitable for use with human embryonic stem cells (ESCs, Biological Industries Inc.) and 2-mercaptoethanol (Millipore). The cells were cultured in suspension and treated with different concentrations of niclosamide for 7 days. The cell culture was examined daily for sphere formation.

### Glucose consumption assay

The CP70 and SKOV3 ovarian cancer cell lines were seeded at a density of 3 × 10^5^ cells per well in 24-well plates with fresh culture medium. Nano-NI was added to each well to obtain a final concentration of 3 μM in each well. The cell viability was also evaluated using the CyQUANT cell proliferation kit assay (Invitrogen), which measures the cellular DNA content via fluorescent dye binding. First, we ensured that the cells were alive for 0 to 4 h (Supplementary Figure S4). The culture medium was collected after 2 and 4 h to check the glucose level under each condition. Fresh medium alone was used in the control group. The glucose level was measured at the Union Clinical Laboratory, Taipei, Taiwan (certified by the College of American Pathologists). Glucose consumption was expressed as the glucose level of the original medium minus that of the sample.

### ATP production assay

The CP70 and SKOV3 ovarian cancer cell lines were seeded at a density of 2 × 10^3^ cells per well in a 96-well plate in fresh culture medium. Next, following a scheduled time course, we added the nano-NI to each well to obtain a final concentration of 3 μM in the test wells. Then, the luminescent cell viability assay kit (Promega) was used to collect the supernatant for laminator analysis, the measurements were performed after 15 min using a microplate reader, and then calculated using the Gen5 software (BioTek).

### Oxygen consumption rate and glycolysis (extracellular acidification rate) test using XF24

Mitochondrial respiration in the CP70 and SKOV3 ovarian cancer cell lines was assessed using a Seahorse XF24 extracellular flux analyzer (Seahorse Bioscience, North Billerica, MA, USA). The CP70 and SKOV3 cells were cultured on Seahorse XF24 plates at a density of 2 × 10^3^ cells/well. Each well was filled with unbuffered RPMI medium at pH 7.4 and incubated at 37°C before the start of the experiment. Baseline measurements of OCR were performed using the oxygen concentration change while the ECAR was measured using the pH change before the wells were injected with nano-NI (final concentration of 3 μM). These agents were injected through the ports of the Seahorse flux pak cartridges to a final concentration of 3 μM. The OCR reading represented the OCR, whereas the ECAR reflected lactate production and was used as an index of glycolysis.

### Xenograft test and positron emission tomography (PET) scan analysis

All procedures were approved by the Institutional Animal Care and Use Committee of the National Defense Medical Center. Female NOD/SCID mice were purchased from the Laboratory Animal Center of the National Taiwan University College of Medicine with health reports confirming that they were free from contamination. Next, 1 × 10^6^ CP70 and SKOV3 cells were inoculated intraperitoneally. Three days later, 100 mg/kg nano-NI was administered to each experimental group, and the same volume of PBS was administered to the control group of NOD/SCID mice five times a week. After 2 weeks of treatment, both groups (*n =* 4) of NOD/SCID mice and a negative control group (*n =* 3) were injected with 0.20–0.25 mCi (7.4–9.25 MBq) of [^18^F]-2-deoxy-2-fluoro-D-glucose ([^18^F]-FDG) via the tail vein.[57] After 30 min to allow the distribution of the [^18^F]-FDG injection, the mice were anesthetized, and animal PET statistic scanning with BIOPET105 (Bioscan, Inc., Washington DC, USA) was performed. The PET images were acquired for 30 min, and the energy window was set at 250–700 keV. The 3D-ordered-subsets expectation maximization (OSEM) was used to reconstruct the images. The entire imaging procedure was performed in the basement of the National Defense Medical Center Laboratory Animal Center, which is certified by the Association for Assessment and Accreditation of Laboratory Animal Care International (AAALAC 2007). Imaging data were analyzed using the AMIDE software v1.0.4 [58]. The tumor volume was quantitated by calculating the SUV, which represents the levels of the [^18^F]-FDG in a volume of interest (typically of a focal tumor) relative to the average [^18^F]-FDG levels in the entire body, detected using animal-PET statistic scanning. The experiment lasted for 3–4 weeks. When the tumor size reached 1.5 cm in diameter in each group, all of the mice were euthanized (approximately 2 weeks after the animal PET scan). The tumor tissue was collected, weighed, and the vital organs including the liver, brain, and kidneys were collected for hematoxylin and eosin (H & E) staining.

### Mouse blood analysis

After 4 weeks, all the mice were euthanized for the analysis of blood biochemistry and tumor sampling. Whole blood was collected via heart puncture, and all of the samples were stored at < 4°C on ice for biochemical analysis. Each whole blood sample (200 μL, *n =* 3) was collected in a K2 EDTA tube via heart puncture after the mice were euthanized. Blood serum was sent for analyses of albumin, alanine aminotransferase (ALT), aspartate aminotransferase (AST), creatinine (Cr), blood urea nitrogen (BUN), and complete blood cell counts to the Taiwan Mouse Clinic, Taiwan.

### Pharmacokinetic animal study

The surgical procedure used here was as described previously [59]. Briefly, female Sprague-Dawley rats were purchased from BioLASCO Taiwan Co. (Taipei, Taiwan, Republic of China) and guaranteed to be specific pathogen free. All the rats were bred and maintained under a 12-h light-dark cycle at a temperature of 21 ± 2°C. Female rats weighing 220–250 g were anesthetized with an intraperitoneal injection of sodium pentobarbital (50 mg/kg). Polyethylene catheters were implanted into the right jugular vein and left carotid artery, and the distal end of the catheter was externalized through an incision in the skin on the back of the neck for drug injection and blood withdrawal (Supplementary Figure S5). The cannulated animals were allowed to recover under normal conditions overnight. The nano-NI was dispersed in PBS (0.46 mg/mL), and administered to rats by oral gavage or IV (5 or 2 mg/kg). The blood samples (150 mL) were collected via the jugular vein cannula at 0 (immediately before dosing) 1, 3, 5, 10, 15, and 30 min, and 1, 2, 4, 6, 8, 12, and 24 h (*n =* 3 and 5 in the IV and oral groups, respectively). Plasma samples were collected by centrifugation (1500 *g* for 15 min at 4°C) and stored at −20°C until the LC-MS/MS analysis was performed (Supplementary information section for details). The AUC from time 0 to infinity (AUC_0-inf_) was calculated as follows: AUC_0-inf_ *=* AUC_0-t_ *+ C*_t_/ λ, where AUC_0-t_ is the AUC from time 0 to the last sampling time (*t*), which is calculated using the trapezoidal rule; *C*_t_/ λ is the AUC extrapolated from *t* to infinity and λ is the terminal rate constant estimated by linear regression to the terminal log-linear portion of the pharmacokinetic profile [29]. Both the oral and IV AUCs (AUC_PO_ and AUC_IV_, respectively) were calculated, and the absolute oral bioavailability (F) was estimated as follows: F (%) *=* (AUC_PO_/AUC_IV_) × (Dose_IV_/Dose_PO_) × 100.

### Statistical analyses

The Student *t*-test (two-tailed) was used to analyze the differences between the nano-NI and control groups in the cell studies. The data are presented as the mean ± SD of the measurements and *p* < 0.05 was considered statistically significant. An unpaired *t*-test was used for the tumor uptake analysis in the animal PET experiment. The GraphPad Prism 5.0 software was used to prepare the illustrations.

## SUPPLEMENTARY MATERIALS FIGURES


