# Supplementary material for: Preclinical evaluation of a nanoformulated antihelminthic, niclosamide, in ovarian cancer
Source: Oncotarget. 2016 Feb 1;7(8):8993–9006. doi: 10.18632/oncotarget.7113 (PMC4891020; doi:10.18632/oncotarget.7113)
Supplement: Supplementary file 1 [file oncotarget-07-8993-s001.pdf]

Preclinical evaluation of a nanoformulated antihelminthic, niclosamide, in ovarian cancer

Supplementary Materials

1. **Liquid chromatography coupled with tandem mass spectrometry (LC-MS/MS) for pharmacokinetic sample analysis**

1.1 Chemicals and reagents

1.2 Niclosamide (99.7%) was purchased from Sigma-Aldrich Chemical Co., Inc. Naproxen (99.5%) was purchased from Sigma-Aldrich Chemical Co., Inc. Chemical structures are shown in Figure 1. Acetonitrile and formic acid, HPLC grade, were obtained from Tedia Company, Inc. (OH, USA). All of the other chemicals were of analytical grade and commercially available. Water was prepared using a Milli-Q water purification system (Millipore, Bedford, MA, USA).

1.3 Instruments

HPLC was performed on an ekspert™ ultraLC 100 system (AB-Sciex; Foster City, CA, USA) with a cooling auto-sampler and a binary solvent delivery pump. The tandem triple-quadrupole mass spectrometer was from API Qtrap 5500 (Applied Biosystems-Sciex; Foster City, CA, USA). Data were acquired and processed using Analyst 1.6.1 software.

1.4 HPLC conditions

An Acquity UPLC™ HSST3 column (100 ×2.1 mm, 1.8 µm; Waters Corp., Milford, MA, USA) was employed for the separation at 40°C. The mobile phase consisted of Solvent A (10 mM ammonium acetate containing 0.1% formic acid) and Solvent B (acetonitrile). The 9-min gradient was as follows: from 0 to 0.5 min, the percentage of eluent B was maintained at 20%; from 0.5 to 5 min, the percentage of eluent B linearly increased from 20% to 90%, and was maintained at 90% for 0.5 min, then returned to 20% in 0.1 min and was maintained at 20% for 3.5 min. The flow rate was set at 0.3 mL/min. The auto-sampler was conditioned at 4°C in this method.

1.5 Mass spectrometry.

A tandem triple-quadrupole mass spectrometry (Applied Biosystems-Sciex API Qtrap5500; Foster City, CA, USA) was operated in negative electrospray ionization (ESI-) mode. The multiple reaction monitoring mode was selected for quantification of the analytes, for which the precursors to production ion transitions were as follows: niclosamide 327→172.9 and naproxen 228.9→185.1. The ion source temperature was maintained at 550°C and the ionspray voltage was –4.5 kV. Analyst®1.6.1 software (Applied Biosystems-Sciex; Foster City, CA, USA) was used.

1.6 Preparation of standards and quality control samples

Standard master stock solutions of niclosamide (1 mg/mL) and naproxen (IS) (1 mg/mL) were obtained by dissolving an appropriate amount of niclosamide and internal standard (IS) in acetonitrile. These stock solutions were serially diluted and added to Sprague-Dawley (SD) rat plasma (ratio 1:9), to produce final concentrations of 1, 5, 10, 50, 100, 250, 500, and 1000 ng/mL for niclosamide. Quality control samples were similarly prepared using a stock solution. The lower limit of quantification and low (L), medium (M), and high (H) quality controls were prepared in plasma at concentrations of 1, 3, 450, and 900 ng/mL, respectively. The IS stock solution containing naproxen (1 mg/mL) was diluted with acetonitrile to prepare a final concentration of 1,000 ng/mL.

1.7 Sample extraction

SD rat plasma (20 µL) was mixed with 200 µL of acetonitrile containing 1,000 ng/mL of naproxen as IS. The mixture was vortexed for 30 s and then centrifuged at 6,000*g* for 5 min in an Eppendorf centrifuge at 4°C. An aliquot of 5 µL of the supernatant was injected into the HPLC-MS/MS system for analysis.

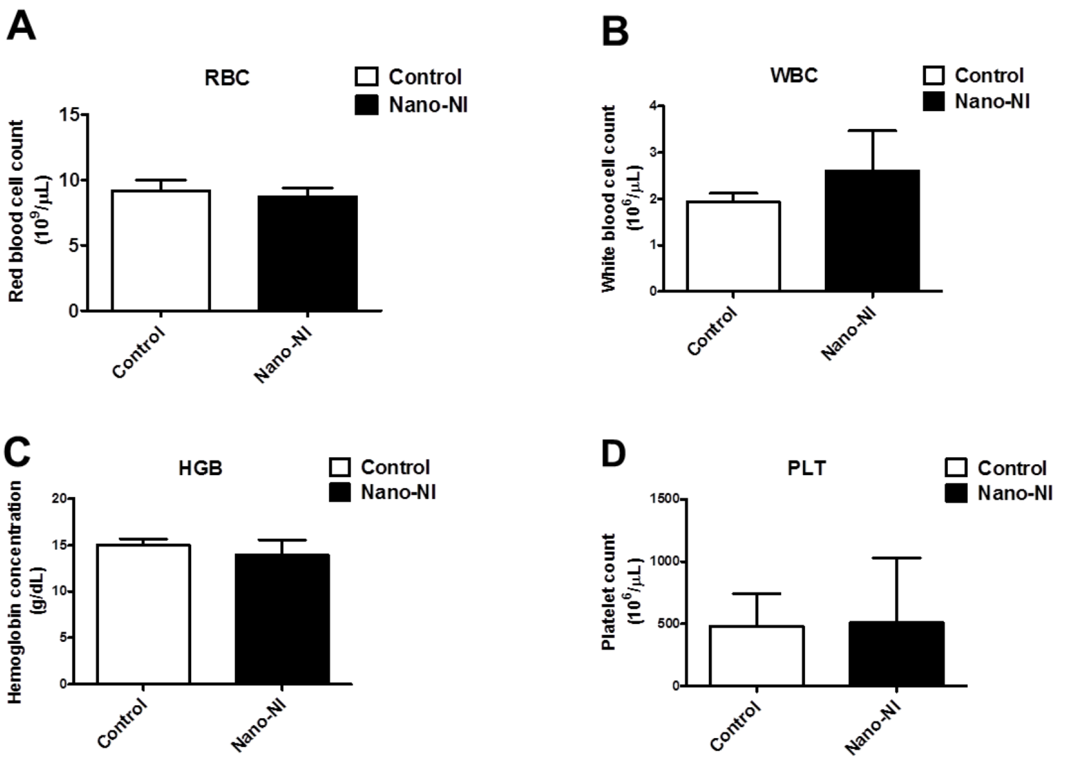

Supplementary Figure S1: Complete blood cell count analysis of both control group (oral phosphate-buffered saline [PBS]) and oral nano-niclosamide (NI) group of non-obese diabetic (NOD)/severe combined immunodeficient (SCID) mice (n = 3, each group). (**A**) Red blood cell counts, RBC (control = $9.2 \pm 0.8 \times 10^{12}$/L, nano-NI = $8.6 \pm 0.7 \times 10^{12}$/L) ($p = 0.25$). (**B**) White blood cell counts, WBC (control = $8.6 + 0.7 \times 10^{9}$/L, nano-NI = $2.6 \pm 0.8 \times 10^{9}$/L) ($p = 0.13$). (**C**) Hemoglobin levels (control = 15 ± 0.7 g/dL, nano-NI = 13.9 ± 1.6 g/dL) ($p = 0.18$). (**D**) Platelet counts (control = $482 \pm 261 \times 10^{9}$/L, nano-NI = $489 \pm 535 \times 10^{9}$/L) ($p = 0.49$).

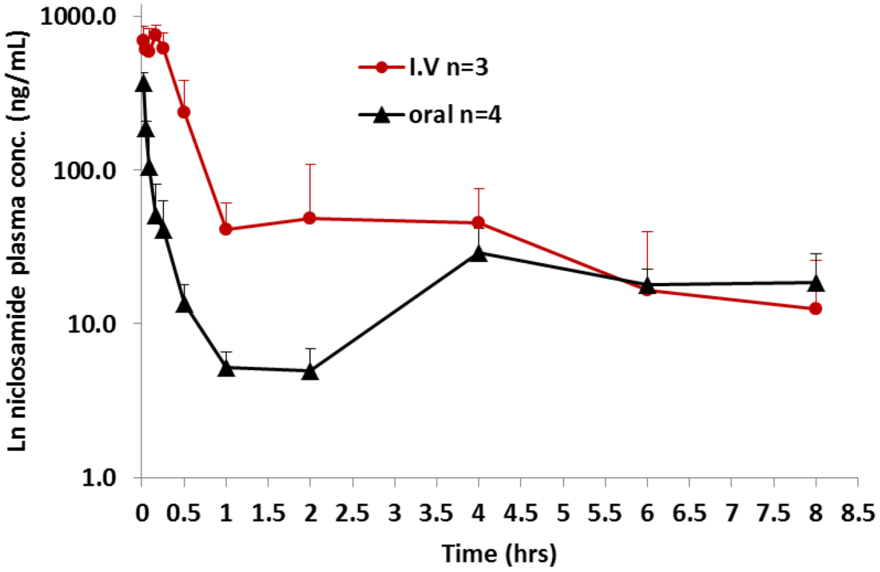

Supplementary Figure S2: Pharmacokinetic analysis of nano-niclosamide following oral and intravenous administration in SD rats (from 0 to 8 h). After oral (5 mg/kg) and intravenous (2 mg/kg) administration of nano-NI, blood samples were collected at 1, 3, 5, 10, 15, and 30 min, and at 1, 2, 4, 6, and 8 h, and were analyzed by LC-MS/MS. The results showed rapid absorption and distribution after oral administration, with a peak concentration at 1 min.

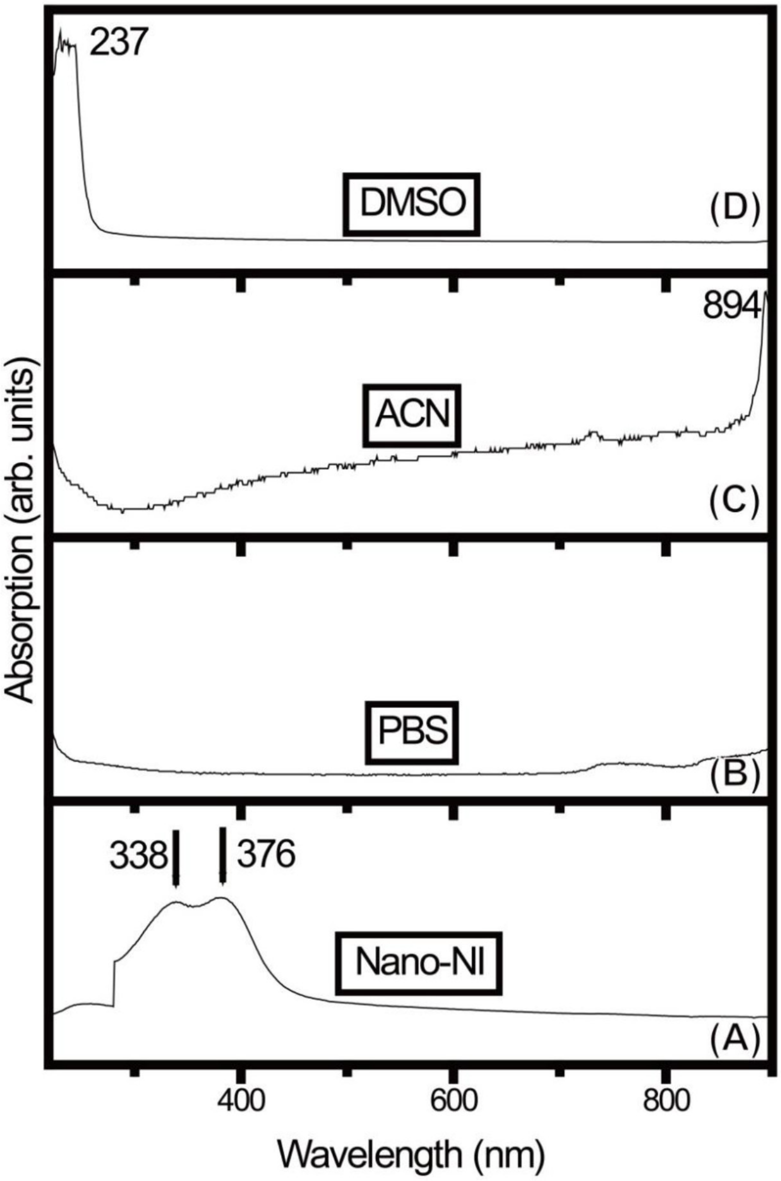

Supplementary Figure S3: UV-visible spectrum of (A) nano-NI, (B) PBS, (C) acetonitrile, and (D) dimethyl sulfoxide. The inset values are the maximum absorption wavelengths for each liquid.

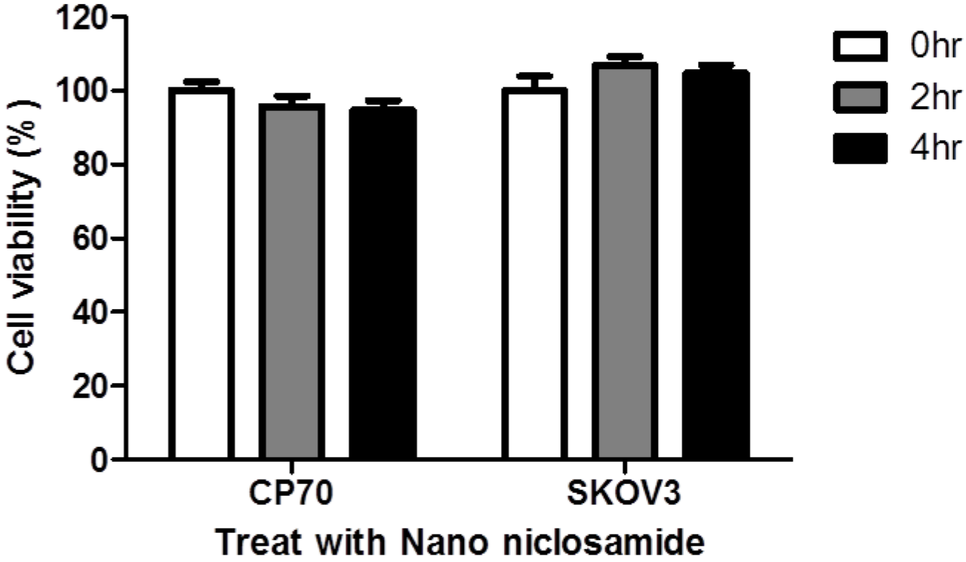

Supplementary Figure S4: Cell viability test after nano-NI treatment in ovarian cancer lines. Cell viability of both ovarian cancer cell lines CP70 and SKOV3 after 0, 2, and 4 h of nano-NI treatment versus the control group using the CyQUANT cell proliferation assay kit.

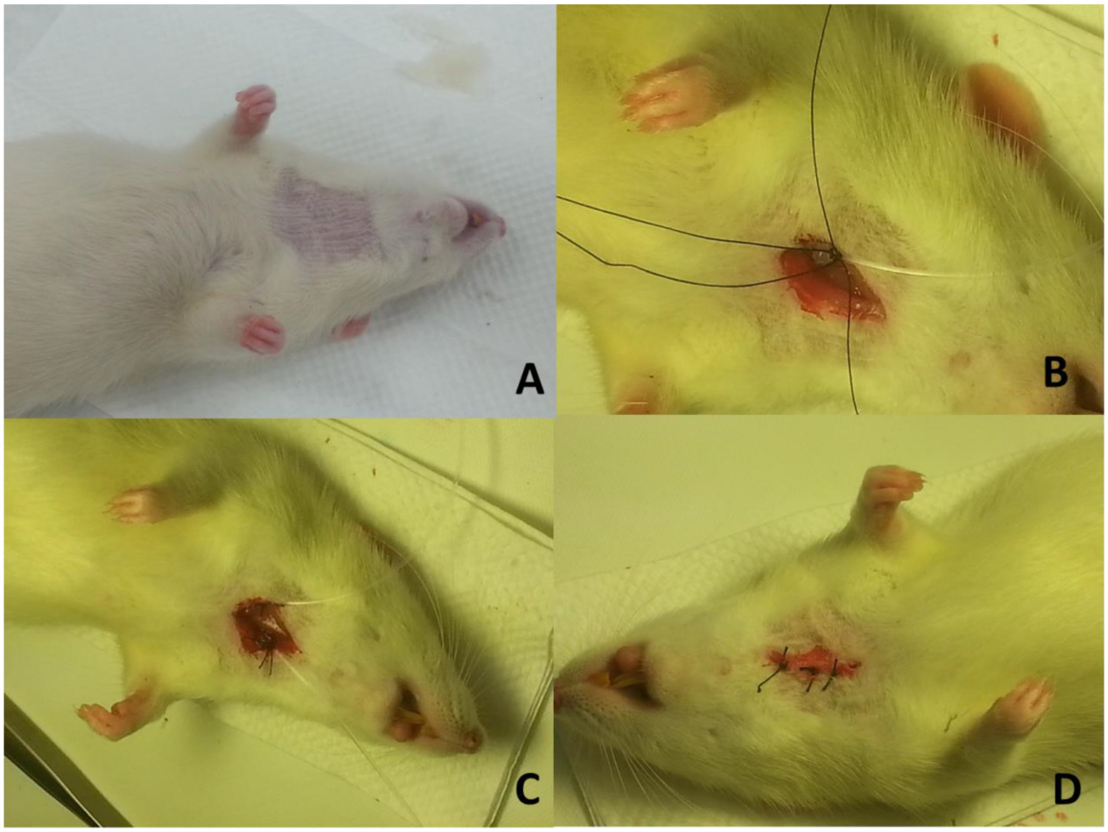

Supplementary Figure S5: Surgical preparation of female SD rats for drug pharmacokinetic experiments. (**A**) Skin prepared for the incision site. (**B, C**) After incision and identification of target vessels, polyethylene catheters were placed in the right jugular vein and left carotid artery and the distal end of the catheter was externalized through an incision in the back of the neck for drug administration and blood withdrawal. (**D**) Skin closed with a Vicryl suture.
